# Loads of trematodes: discovering hidden diversity of paramphistomoids in Kenyan ruminants

**DOI:** 10.1017/S0031182016001827

**Published:** 2016-10-20

**Authors:** MARTINA R. LAIDEMITT, EVA T. ZAWADZKI, SARA V. BRANT, MARTIN W. MUTUKU, GERALD M. MKOJI, ERIC S. LOKER

**Affiliations:** 1 Department of Biology, Center for Evolutionary and Theoretical Immunology, Parasite Division Museum of Southwestern Biology, University of New Mexico, 167 Castetter MSCO3 2020 Albuquerque, New Mexico 87131, USA; 2 Center for Biotechnology Research and Development, Kenya Medical Research Institute (KEMRI), P.O. Box 54840-00200, Nairobi, Kenya

**Keywords:** Paramphistomoidea, biodiversity, DNA barcode, host specificity, *Schistosoma*

## Abstract

Paramphistomoids are ubiquitous and widespread digeneans that infect a diverse range of definitive hosts, being particularly speciose in ruminants. We collected adult worms from cattle, goats and sheep from slaughterhouses, and cercariae from freshwater snails from ten localities in Central and West Kenya. We sequenced *cox*1 (690 bp) and internal transcribed region 2 (ITS2) (385 bp) genes from a small piece of 79 different adult worms and stained and mounted the remaining worm bodies for comparisons with available descriptions. We also sequenced *cox*1 and ITS2 from 41 cercariae/rediae samples collected from four different genera of planorbid snails. Combining morphological observations, host use information, genetic distance values and phylogenetic methods, we delineated 16 distinct clades of paramphistomoids. For four of the 16 clades, sequences from adult worms and cercariae/rediae matched, providing an independent assessment for their life cycles. Much work is yet to be done to resolve fully the relationships among paramphistomoids, but some correspondence between sequence- and anatomically based classifications were noted. Paramphistomoids of domestic ruminants provide one of the most abundant sources of parasitic flatworm biomass, and because of the predilection of several species use *Bulinus* and *Biomphalaria* snail hosts, have interesting linkages with the biology of animal and human schistosomes to in Africa.

## INTRODUCTION

The Superfamily Paramphistomoidea is a prominent group of digeneans where adults are characterized by the absence of an oral sucker and the presence of an acetabulum at or near the posterior end of the body. The systematics of this group of digeneans is a work in progress. Sey (1991) concluded it is comprised of eight families, whereas Jones (2005*a*) concluded there are 12 families. Paramphistomoids are often called rumen flukes because many of the best-known representatives live in this habitat in domestic ruminants. However, many species also inhabit the intestines of fish, amphibians, reptiles, birds and non-ruminant mammals. They feature a life cycle in which cercariae produced in rediae emerge from snails and encyst on vegetation as metacercariae, which are later ingested by the definitive host (Jones, 2005*a*). As part of a larger study to determine how digenean community diversity influences the transmission of schistosomes in Kenya, we provide new results regarding the overall diversity and host relationships of paramphistomoids in Kenya, based on cercariae collected from snails and adult worms from domestic animals from abattoirs.

Paramphistomoids are of interest to parasitologists in several contexts. They are diverse in number of species and provide an understudied model group for those focused on revealing patterns and mechanisms of diversity. Of the 12 recognized paramphistomoid families recognized by Jones (2005*a*), representatives of nine occur in Africa. The diversity of paramphistomoids in Africa reflects the presence of many species of terrestrial mammals, including elephants, rhinoceroses, hippopotami and a rich diversity of wild and domestic ruminants. Three families in particular (Paramphistomidae, Gastrodiscidae and Gastrothylacidae) are speciose in Africa. The distribution of diversity in rumen hosts can partly be explained by characters (e.g. regressed pharyngeal appendages) that are apomorphic, which have allowed them to colonize the forestomach (Sey 1991). The three families comprise over 40% of all known paramphistomoids, the majority of which use ruminants as their definitive hosts (Sey, 1991).

Paramphistomoids have thick bodies, which make detailed morphological characterization of adult features and species identification challenging (Horak, 1971; Jones, 1991; Mage *et al.* 2002; Rinaldi *et al.* 2005). The bodies of paramphistomoid cercariae are also relatively thick and typically filled with cystogenous material or pigment, also rendering identification difficult. Nonetheless, a meticulous framework for paramphistomoid identification and classification has been developed (see reviews by Sey, 1991; Jones, 2005*a*). Given the inherent difficulties in identification, coupled with a growing list of studies from other digenean groups documenting the presence of cryptic species (Detwiler *et al.* 2012; Herrmann *et al.* 2014; McNamara *et al.* 2014), paramphistomoids are ideal for studies attempting to meld traditional morphological identification with sequence data characterization provided by molecular approaches. The number of studies that use molecular techniques to provide assessments of the diversity of paramphistomoids have in general been limited, especially so for African species (Lotfy *et al.* 2010; Mansour *et al.* 2014; Sibula *et al*. 2014; Titi *et al.* 2014; Dube *et al.* 2015).

In addition to being speciose, paramphistomoids are often remarkably abundant (Horak, 1971; Cheruiyot and Wamae, 1988; Rolfe *et al.* 1994; Sanabria and Romero, 2008). In fact, one might be hard pressed to find a larger source of sheer digenean biomass than is presented routinely at abattoirs by ruminant paramphistomoids. Given the large worm populations that can occur in individual cattle, goats or sheep, vast numbers of paramphistomoid eggs are regularly passed into the environment. In rural West Kenya, we can routinely collect 10 000 paramphistomoid eggs from a single cow dung sample. As domestic ruminants regularly seek water from natural habitats, it is not surprising that many paramphistomoid eggs enter freshwater, creating the potential for high levels of infection in their snail hosts (Chingwena *et al.* 2002*a*; Mohammed *et al.* 2016).

A review of the East African paramphistomoid literature reveals that many of the described species are transmitted by *Biomphalaria* and *Bulinus*, the snail genera also of concern with respect to their role in transmission of human schistosomiasis in Africa (Dinnik, 1954; Dinnik and Dinnik, 1957; Dinnik, 1961; Eduardo, 1983; Brown, 1994; Chingwena *et al.* 2002*b*; Jones, 2005*b*, *c*). In some areas, *Bulinus* and *Biomphalaria* are the most commonly implicated snail hosts for paramphistomoids (Dinnik, 1965; Wright *et al.* 1979; Loker *et al.* 1981; Chingwena *et al.* 2002*b*; Ahmed *et al.* 2006; Mohammed *et al*. 2016). The presence of other digenean species utilizing the same snail species as schistosomes could be a factor that influences the overall success of animal and human schistosome transmission (Lim and Heyneman, 1972; Combes, 1982; Hechinger *et al.* 2011; Spatz *et al.* 2012). This is particularly so for species such as paramphistomoids that produce rediae as larval stages within their snail hosts, because rediae may attack, damage and consume schistosome sporocysts (Lim and Heyneman, 1972).

We collected cercariae and adult worms from ten localities in Kenya. We provide stained whole mounts and provisional identification of adults that are linked to sequence data for cytochrome oxidase 1 (*cox*1) and the internal transcribed region 2 (ITS2). In some cases, we provide matches with sequences obtained from cercariae and adult worms thus providing probable life cycle linkages. We also provide new hypotheses for phylogenetic relationships among the paramphistomoids that include available sequences from NCBI GenBank, which show that some species of paramphistomoids are geographically widespread throughout Africa. Data presented here will contribute to an increased understanding of the superfamily Paramphistomoidea, including providing greater clarification for how these worms are distributed among hosts, their potential roles if any in causing disease in domestic or wild animals, and their interactions with other digeneans, including schistosomes.

## MATERIALS AND METHODS

### Sampling

We collected larval and adult paramphistomoids from ten different localities in central and especially western Kenya between 2005 and 2015 (Table 1). All species of field-collected aquatic snails were brought to the laboratory at Kisian, near Kisumu, Kenya. The snails were cleaned and then placed individually into 12-well tissue culture plates in 3 mL of aged tap water. The tissue culture plates were placed in natural light for 2 h to induce shedding of cercariae. Snails shedding cercariae were identified using keys and information in Brown and Kristensen (1989) and Brown (1994), and cercariae were preliminarily identified using keys (Frandsen and Christensen, 1984; Schell, 1985) and by reference to regional monographs (e.g. Fain, 1953). All cercariae designated as paramphistomoids were confirmed as such according to Sey (1991). Snails were either dissected at the time of collection to procure rediae, or re-shed two and four weeks later to determine if snails were harboring prepatent infections at the time of collection. Snails were kept in 20 L plastic tanks and fed red leaf lettuce following collection. Cercariae and rediae were preserved in 95% ethanol for later molecular analysis.

**Table 1.** Collection localities in central and west Kenya

Site name	Lat.	Long.
Asao Stream	−0·3181	35·0069
Katito Slaughterhouse	−0·2700	34·9719
Sondu Slaughterhouse	−0·3927	35·018
Kasabong Stream	−0·1519	34·3355
Mgosi Slaughterhouse	−0·0768	34·7754
Mwea	−0·8180	37·6220
Ng'alalia	−1·5357	37·2361
Kibwezi Slaughterhouse	−2·4167	37·9667
Nyabera Swamp	−0·1091	34·7750
Powerhouse Lake Victoria	−0·0941	34·7076

Adults were collected from the rumen or reticulum of *Bos indicus, Capra aegagrus hircus* and *Ovis aries* from one slaughterhouse in central Kenya and three in Western Kenya (Table 1). Adults were preserved in 95% ethanol for later molecular and morphological identification.

### Staining adult worms

Adult worms were placed into 70% ethanol for 24 h prior to staining. Sections of the adult worms were stained and mounted according to Eduardo (1982). Because of their thickness, each adult was sectioned frontally using a razor blade. Part of the posteroterminally placed acetabulum was severed and used for molecular analysis.

### Collection of molecular data

A partial sequence of *cox*1 mtDNA and internal transcribed spacer two (ITS2) were amplified by polymerase chain reaction (PCR) to facilitate differentiation among paramphistomoid specimens. One to six cercariae, one to three rediae or a portion of the acetabulum from adults were used for DNA extraction. Genomic DNA was extracted from 120 paramphistomoid samples (Table 2) by the alkaline-lysis (HOT-SHOT) method (Truett *et al.* 2000), or by the QIAamp DNA Micro Kit following the manufacturer's instructions, with a final elution volume of 30 *µ*L (Qiagen, Valencia, CA). Although not the equal of the QIAamp Kit with respect to absolute quality of the DNA produced, the HOT-SHOT method also produced DNA of quality and proved more amenable for use under conditions where controlled conditions were less available.

**Table 2.** Specimen name, host collected from, collection locality, provisional identification, Museum of Southwestern Biology/KEMRI voucher numbers, and GenBank accession numbers of paramphistomoid specimens used in this study

Specimen name host	Provisional ID	Stage	Locality	Year	MSB/KEMRI Voucher	GenBank ITS2	GenBank *cox*1
PA1 Goat	*Calicophoron microbothrium*	Adult	Asao Stream	Aug-12	MSB:Para:25079	KX668901	KX670098
PA2 Cattle	*Cotylophoron* sp.	Adult	Mgosi	Feb-13	MSB:Para:25101	KX668933	KX670128
PA3 Cattle	*Calicophoron clavula*	Adult	Mgosi	Jan-10	MSB:Para:25088	KX668944	KX670139
PA4 Sheep	*Calicophoron raja*	Adult	Mgosi	Feb-13	MSB:Para:25078	KX668955	KX670150
PA5 Cattle	*Calicophoron raja*	Adult	Mgosi	Oct-13	MSB:Para:25051	KX668966	KX670161
PA6 Goat	*Calicophoron phillerouxi*	Adult	Asao Stream	Aug-12	MSB:Para:25080	KX668977	KX670172
PA7 Goat	*Calicophoron microbothrium*	Adult	Mgosi	Oct-13	MSB:Para:25050	KX668988	KX670183
PA8 Sheep	Paramphistomoidea	Adult	Mgosi	Nov-13	MSB:Para:25047	KX668999	KX670194
PA9 Sheep	Paramphistomoidea	Adult	Mgosi	Dec-13	MSB:Para:25053	KX669010	KX670205
PA10 Cattle	*Carmyerius mancupatus*	Adult	Mgosi	Jan-14	*MSB:Para:25300/KEMRI:Para:1	KX668902	KX670099
PA11 Cattle	*Cotylophoron* sp.	Adult	Mgosi	Jan-14	*MSB:Para:25045/KEMRI:Para:2	KX668913	KX670108
PA12 Cattle	*Carmyerius gregarius*	Adult	Mgosi	Jan-14	*MSB:Para:25055/KEMRI:Para:3	KX668924	KX670119
PA13 Goat	*Cotylophoron* sp.	Adult	Mgosi	Jan-14	*MSB:Para:25157//KEMRI:Para:4	KX668926	KX670121
PA14 Sheep	*Calicophoron raja*	Adult	Mgosi	Jan-14	*MSB:Para:25153/KEMRI:Para:5	KX668927	KX670122
PA15 *Ceratophallus* *natalensis*	Paramphistomoidea	Cercariae	Nyabera	Jan-05	MSB:Para:25059	KX668928	KX670123
PA16 *Ceratophallus* *natalensis*	Paramphistomoidea	Cercariae	Nyabera	Jan-05	MSB:Para:25060	KX668929	KX670124
PA17 *Biomphalaria* *pfeifferi*	Paramphistomoidea	Cercariae	Kasabong	Jan-14	*MSB:Para:25138/KEMRI:Para:6	KX668930	KX670125
PA18 *Biomphalaria* *pfeifferi*	Paramphistomoidea	Cercariae	Asao Stream	Feb-13	MSB:Para:25065	KX668931	KX670126
PA19 *Biomphalaria* *pfeifferi*	Paramphistomoidea	Cercariae	Asao Stream	Jan-15	*MSB:Para:25287/KEMRI:Para:7	KX668932	KX670127
PA20 *Biomphalaria* *pfeifferi*	Paramphistomoidea	Cercariae	Asao Stream	Jan-15	*MSB:Para:25288/KEMRI:Para:8	KX668934	KX670129
PA21 *Bulinus* *forskalii*	*Calicophoron phillerouxi*	Cercariae	Mwea	Feb-13	MSB:Para:25064	KX668935	KX670130
PA22 *Bulinus* *forskalii*	*Calicophoron microbothrium*	Cercariae	Ng'alalia	May-10	MSB:Para:25150	KX668936	KX670131
PA23 *Biomphalaria* *pfeifferi*	Unknown	Cercariae	Asao Stream	Jul-15	*MSB:Para:25289/KEMRI:Para:9	KX668937	KX670132
PA24 Cattle	*Carmyerius gregarius*	Adult	Mgosi	May-10	MSB:Para:25113	KX668938	KX670133
PA25 Cattle	*Carmyerius mancupatus*	Adult	Mgosi	Jun-14	*MSB:Para:25070/KEMRI:Para:10	KX668939	KX670134
PA26 Cattle	*Carmyerius exporous*	Adult	Mgosi	Jun-14	*MSB:Para:25071/KEMRI:Para:11	KX668940	KX670135
PA27 Cattle	*Calicophoron microbothrium*	Adult	Mgosi	Jun-14	*MSB:Para:25073/KEMRI:Para:12	KX668941	KX670136
PA28 Cattle	*Calicophoron raja*	Adult	Mgosi	Jan-10	MSB:Para:25085	KX668942	KX670137
PA29 Cattle	*Calicophoron microbothrium*	Adult	Kibwezi	Oct-13	MSB:Para:25092	KX668943	KX670138
PA30 Cattle	*Calicophoron microbothrium*	Adult	Kibwezi	Oct-13	MSB:Para:25093	KX668945	KX670140
PA31 *Segmentorbis*	Gastrothylacidae	Cercariae	Lake Victoria	Oct-13	MSB:Para:25094	KX668946	KX670141
PA32 *Segmentorbis*	Gastrothylacidae	Cercariae	Lake Victoria	Oct-13	MSB:Para:25095	KX668947	KX670142
PA33 Cattle	*Carmyerius exporous*	Adult	Mgosi	Jan-10	MSB:Para:25114	KX668948	KX670143
PA34 *Segmentorbis*	Gastrothylacidae	Cercariae	Lake Victoria	Oct-13	MSB:Para:25096	KX668949	KX670144
PA35 Cattle	*Calicophoron microbothrium*	Adult	Mgosi	Jan-10	MSB:Para:25115	KX668950	KX670145
PA36 *Ceratophallus* *natalensis*	*Carmyerius mancupatus*	Cercariae	Nyabera	Jan-15	*MSB:Para:25290/KEMRI:Para:13	KX668951	KX670146
PA37 Cattle	*Cotylophoron* sp.	Adult	Mgosi	Feb-13	MSB:Para:25109	KX668952	KX670147
PA38 Cattle	*Carmyerius exporous*	Adult	Mgosi	Feb-13	MSB:Para:25145	KX668953	KX670148
PA39 Cattle	*Calicophoron phillerouxi*	Adult	Mgosi	Feb-13	MSB:Para:25108	KX668954	KX670149
PA40 Cattle	*Calicophoron clavula*	Adult	Mgosi	Jan-10	MSB:Para:25081	KX668956	KX670151
PA41 Cattle	*Calicophoron microbothrium*	Adult	Mgosi	Jan-14	*MSB:Para:25048/KEMRI:Para:14	KX668957	KX670152
PA42 Cattle	*Cotylophoron* sp.	Adult	Mgosi	Jan-14	*MSB:Para:25054/KEMRI:Para:15	KX668958	KX670153
PA43 Cattle	*Cotylophoron cotylophorum*	Adult	Mgosi	Jan-10	MSB:Para:25083	KX668959	KX670154
PN1 Notocotylidae	Notocotylidae	Cercariae	Lake Victoria	Jan-15	*MSB:Para:25299/KEMRI:Para:16	KX669021	KX670216
PA44 Goat	*Cotylophoron* sp.	Adult	Mgosi	Jan-14	*MSB:Para:25302/KEMRI:Para:17	KX668960	KX670155
PA45 Cattle	*Calicophoron phillerouxi*	Adult	Mgosi	Oct-13	MSB:Para:25057	KX668961	KX670156
PA46 Cattle	*Calicophoron phillerouxi*	Adult	Katito	Mar-13	MSB:Para:25142	KX668962	KX670157
PA47 Sheep	*Calicophoron phillerouxi*	Adult	Mgosi	Jan-13	MSB:Para:25105	KX668963	KX670158
PA48 Goat	*Calicophoron phillerouxi*	Adult	Asao	Aug-12	MSB:Para:25075	KX668964	KX670159
PA49 Goat	*Calicophoron phillerouxi*	Adult	Asao	Aug-12	MSB:Para:25076	KX668965	KX670160
PA50 *Biomphalaria* *pfeifferi*	Paramphistomoidea	Cercariae	Kasabong	Jan-15	*MSB:Para:25291/KEMRI:Para:18	KX668967	KX670162
PA51 Cattle	*Cotylophoron* sp.	Adult	Mgosi	Feb-13	MSB:Para:25107	KX668968	KX670163
PA52 Cattle	*Cotylophoron* sp.	Adult	Katito	Feb-13	MSB:Para:25144	KX668969	KX670164
PA53 Sheep	*Cotylophoron* sp.	Adult	Sondu	Feb-13	MSB:Para:25058	KX668970	KX670165
PA54 Goat	*Cotylophoron* sp.	Adult	Mgosi	Feb-13	MSB:Para:25102	KX668971	KX670166
PA55 Cattle	*Cotylophoron* sp.	Adult	Mgosi	Feb-13	MSB:Para:25041	KX668972	KX670167
PA56 Cattle	*Cotylophoron* sp.	Adult	Mgosi	Feb-13	MSB:Para:25074	KX668973	KX670168
PA57 Cattle	*Cotylophoron* sp.	Adult	Mgosi	Feb-13	MSB:Para:25099	KX668974	KX670169
PA58 Cattle	*Cotylophoron* sp.	Adult	Mgosi	Jan-14	*MSB:Para:25292/KEMRI:Para:28	KX668975	KX670170
PA59 Cattle	*Cotylophoron* sp.	Adult	Mgosi	Feb-13	MSB:Para:25106	KX668976	KX670171
PA60 *Segmentorbis*	Gastrothylacidae	Cercariae	Lake Victoria	Oct-13	MSB:Para:25097	KX668978	KX670173
PA61 Cattle	*Carmyerius exporous*	Adult	Katito	Feb-13	MSB:Para:25143	KX668979	KX670174
PA62 Cattle	*Carmyerius exporous*	Adult	Katito	Feb-13	MSB:Para:25110	KX668980	KX670175
PA63 Cattle	*Carmyerius exporous*	Adult	Mgosi	Jun-14	*MSB:Para:25069/KEMRI:Para:33	KX668981	KX670176
PA64 Cattle	*Carmyerius exporous*	Adult	Katito	Feb-13	MSB:Para:25038	KX668982	KX670177
PA65 Cattle	*Carmyerius exporous*	Adult	Katito	Feb-13	MSB:Para:25036	KX668983	KX670178
PA66 Cattle	*Carmyerius exporous*	Adult	Katito	Feb-13	MSB:Para:25112	KX668984	KX670179
PA67 Cattle	*Carmyerius exporous*	Adult	Katito	Feb-13	MSB:Para:25140	KX668985	KX670180
PA68 *Biomphalaria* *pfeifferi*	Paramphistomoidea	Cercariae	Asao	Mar-13	MSB:Para:25135	KX668986	KX670181
PA69 *Biomphalaria* *pfeifferi*	Paramphistomoidea	Cercariae	Kasabong	Mar-13	MSB:Para:25293	KX668987	KX670182
PA70 *Biomphalaria* *pfeifferi*	Paramphistomoidea	Cercariae	Kasabong	Jun-14	*MSB:Para:25297/KEMRI:Para:34	KX668989	KX670184
PA71 *Biomphalaria* *pfeifferi*	Paramphistomoidea	Cercariae	Asao	Mar-13	MSB:Para:25120	KX668990	KX670185
PA72 *Biomphalaria* *pfeifferi*	Paramphistomoidea	Cercariae	Asao	Mar-13	MSB:Para:25124	KX668991	KX670186
PA73 *Biomphalaria* *pfeifferi*	Paramphistomoidea	Cercariae	Asao	Aug-12	MSB:Para:25068	KX668992	KX670187
PA74 *Biomphalaria* *pfeifferi*	Paramphistomoidea	Cercariae	Asao	Aug-12	MSB:Para:25116	KX668993	KX670188
PA75 *Biomphalaria* *pfeifferi*	Paramphistomoidea	Cercariae	Asao	Mar-13	MSB:Para:25121	KX668994	KX670189
PA76 *Biomphalaria* *pfeifferi*	Paramphistomoidea	Cercariae	Kasabong	Jul-14	*MSB:Para:25294/KEMRI:Para:35	KX668995	KX670190
PA77 *Biomphalaria* *pfeifferi*	Paramphistomoidea	Cercariae	Asao	Mar-13	MSB:Para:25118	KX668996	KX670191
PA78 *Biomphalaria* *pfeifferi*	Paramphistomoidea	Cercariae	Asao	Mar-13	MSB:Para:25134	KX668997	KX670192
PA79 *Biomphalaria* *pfeifferi*	Paramphistomoidea	Cercariae	Asao	Feb-13	MSB:Para:25295	KX668998	KX670193
PA80 *Biomphalaria* *pfeifferi*	Paramphistomoidea	Cercariae	Kasabong	Jan-15	*MSB:Para:25296/KEMRI:Para:19	KX669000	KX670195
PA81 *Biomphalaria* *pfeifferi*	Paramphistomoidea	Cercariae	Asao	Mar-13	MSB:Para:25136	KX669001	KX670196
PA82 *Biomphalaria* *pfeifferi*	Paramphistomoidea	Cercariae	Asao	Mar-13	MSB:Para:25137	KX669002	KX670197
PA83 *Biomphalaria* *pfeifferi*	Paramphistomoidea	Cercariae	Asao	Mar-13	MSB:Para:25119	KX669003	KX670198
PA84 *Biomphalaria* *pfeifferi*	Paramphistomoidea	Cercariae	Asao	Feb-13	MSB:Para:25063	KX669004	KX670199
PA85 *Biomphalaria* *pfeifferi*	Paramphistomoidea	Cercariae	Asao	Mar-13	MSB:Para:25122	KX669005	KX670200
PA86 *Biomphalaria* *pfeifferi*	Paramphistomoidea	Cercariae	Asao	Mar-13	MSB:Para:25123	KX669006	KX670201
PA87 *Biomphalaria* *pfeifferi*	Paramphistomoidea	Cercariae	Asao	Mar-13	MSB:Para:25125	KX669007	KX670202
PA88 *Biomphalaria* *pfeifferi*	Paramphistomoidea	Cercariae	Asao	Mar-13	MSB:Para:25126	KX669008	KX670203
PA89 *Biomphalaria* *pfeifferi*	Paramphistomoidea	Cercariae	Asao	Mar-13	MSB:Para:25127	KX669009	KX670204
PA90 *Biomphalaria* *pfeifferi*	Paramphistomoidea	Cercariae	Asao	Mar-13	MSB:Para:25128	KX669011	KX670206
PA91 *Biomphalaria* *pfeifferi*	Paramphistomoidea	Cercariae	Asao	Mar-13	MSB:Para:25130	KX669012	KX670207
PA92 Cattle	*Carmyerius exporous*	Adult	Sondu	Feb-13	MSB:Para:25301	KX669013	KX670208
PA93 Cattle	*Carmyerius exporous*	Adult	Sondu	Feb-13	MSB:Para:25141	KX669014	KX670209
PA94 Cattle	*Carmyerius exporous*	Adult	Sondu	Feb-13	MSB:Para:25151	KX669015	KX670210
PA95 Cattle	*Carmyerius exporous*	Adult	Katito	Feb-13	MSB:Para:25043	KX669016	KX670211
PA96 Cattle	*Carmyerius exporous*	Adult	Sondu	Feb-13	MSB:Para:25111	KX669017	KX670212
PA97 Cattle	*Carmyerius exporous*	Adult	Katito	Feb-13	MSB:Para:25037	KX669018	KX670213
PA98 Cattle	*Carmyerius exporous*	Adult	Sondu	Feb-13	MSB:Para:25044	KX669019	KX670214
PA99 *Biomphalaria* *pfeifferi*	Paramphistomoidea	Cercariae	Kasabong	Jun-14	*MSB:Para:25298/KEMRI:Para:20	KX669020	KX670215
PA100 Sheep	*Carmyerius mancupatus*	Adult	Sondu	Oct-13	MSB:Para:25154	KX668903	KX670100
PA101 Goat	*Carmyerius mancupatus*	Adult	Mgosi	Jan-14	*MSB:Para:25067/KEMRI:Para:21	KX668904	KX670101
PA102 Goat	*Calicophoron raja*	Adult	Mgosi	Jan-14	*MSB:Para:25052/KEMRI:Para:22	KX668905	KX670102
PA103 Goat	*Calicophoron raja*	Adult	Mgosi	Jan-14	*MSB:Para:25046/KEMRI:Para:23	KX668906	KX670103
PA104 Cattle	*Calicophoron raja*	Adult	Mgosi	Jan-14	*MSB:Para:25100/KEMRI:Para:24	KX668907	KX670104
PA105 Cattle	*Calicophoron raja*	Adult	Mgosi	Jan-14	*MSB:Para:25281/KEMRI:Para:25	KX668908	KX670105
PA106 Goat	*Calicophoron raja*	Adult	Asao	Jan-14	*MSB:Para:25077/KEMRI:Para:26	KX668909	KX670106
PA107 Sheep	*Calicophoron microbothrium*	Adult	Katito	Jan-14	*MSB:Para:25049/KEMRI:Para:27	KX668910	KX670107
PA108 Cattle	*Calicophoron microbothrium*	Adult	Sondu	Feb-13	MSB:Para:25039	KX668911	KX670096
PA109 Cattle	*Calicophoron microbothrium*	Adult	Mgosi	Feb-13	MSB:Para:25282	KX668912	KX670097
PA110 Cattle	*Calicophoron microbothrium*	Adult	Mgosi	Jan-10	MSB:Para:25089	KX668914	KX670109
PA111 Cattle	*Calicophoron microbothrium*	Adult	Mgosi	Jan-14	*MSB:Para:25139/KEMRI:Para:29	KX668915	KX670110
PA112 *Bulinus* *forskalii*	*Calicophoron microbothrium*	Cercariae	Kasabong	Jan-15	*MSB:Para:25283/KEMRI:Para:36	KX668916	KX670111
PA113 Cattle	*Calicophoron microbothrium*	Adult	Kibewze	Oct-13	MSB:Para:25091	KX668917	KX670112
PA114 Goat	*Calicophoron microbothrium*	Adult	Mgosi	Jan-14	*MSB:Para:25284/KEMRI:Para:30	KX668918	KX670113
PA115 Goat	*Calicophoron microbothrium*	Adult	Mgosi	Jan-14	*MSB:Para:25056/KEMRI:Para:31	KX668919	KX670114
PA116 Cattle	*Calicophoron microbothrium*	Adult	Mgosi	Jan-14	*MSB:Para:25285/KEMRI:Para:32	KX668920	KX670115
PA117 Sheep	*Calicophoron microbothrium*	Adult	Katito	Feb-13	MSB:Para:25152	KX668921	KX670116
PA118 Goat	*Calicophoron microbothrium*	Adult	Mgosi	Jan-10	MSB:Para:25090	KX668922	KX670117
PA119 Cattle	*Calicophoron microbothrium*	Adult	Sondu	Feb-13	MSB:Para:25286	KX668923	KX670118
PA120 Cattle	*Calicophoron microbothrium*	Adult	Sondu	Feb-13	MSB:Para:25040	KX668925	KX670120

PA1-PA44 contain representatives of the 16 different clades used to construct the ML and Bayesian trees. PA45-PA120 were included in the preliminary trees. An (*) denotes samples that are in Kenya.

*Cox*1 oligonucleotide primers were designed based on the barcode region (Folmer *et al.* 1994) and on conserved regions in the *Fasciola hepatica* (NC_002546), *Paragonimus westermani* (AF219379) and *Paramphistomum cervi* (NC_023095) mitochondrial genomes. *Cox*1 was amplified using primers 123F [5′-ATTCGTTTGAACTATATGGA-3′] and 858R [5′-CATATGATGAGCCCAAACAAC-3′]. The volume of each PCR reaction was 25 *µ*L with 1 *µ*L of 100 ng of DNA, 0·8 mm L^−1^ dNTPs, 2·5 mm L^−1^ MgCl2, 0·25 units of Ex Taq DNA (Clontech, Mountain View, CA) and 0·4 *µ*m L of each primer. PCR cycles were programmed as follows: 2 min denaturation hold at 94 °C; 94 °C for 1 min, 46 °C for 30 s and 72 °C for 1 min for three cycles; 94 °C for 1 min, 45 °C for 30 s, and 72 °C for 1 min for three cycles; 94 °C for 1 min, 44 °C for 30 s and 72 °C for 1 min for three cycles; 94 °C for 1 min, 44 °C for 30 s and 72 °C for 1 min for 20 cycles, and followed by an extension step for 7 min at 72 °C.

ITS2 was amplified using GA1 [5′-AGA ACA TCG ACA TCT TGA AC-3′] (Anderson and Barker, 1998) and BD2 primers [5′-TAT GCT TAA ATT CAG CGG GT-3′] (Bowles *et al.* 1995). The volume of each reaction was 25 *µ*L, with 12·5 *µ*L of Premix Taq™ (Clontech, Mountain View, CA), 0·4 *µ*m L^−1^ of each primer, and one *μ*L of 55 ng of DNA. PCR cycles were performed on Eppendorf Mastercycler epigradient machines, which were programmed as follows: 1 C s^−1^ rate of change, one cycle at 98 °C for 10 s, followed by 30 cycles of 98 °C for 1 min, 52 °C for 2 min and 72 °C for 1 min with an extension step for 7 min at 72 °C.

PCR fragments were separated by agarose gel electrophoresis and visualized with 0·5% GelRed™ Nucleic acid gel stain (Biotium, Hayward, CA). PCR products were purified using the QIAquick purification kit (Qiagen, Valencia, CA) or by ExoSap-IT^®^ (Affymetrix, Santa Clara, CA). Both strands were sequenced using an Applied Biosystems 3130 automated sequencer and BigDye terminator cycle sequencing kit Version 3.1 (Applied Biosystems, Foster City, CA). DNA sequences were verified by aligning reads from the 5′ and 3′ directions using Sequencher 5·0 and manually corrected for ambiguous base calls (Gene Codes, Ann Arbor, MI).

### Outgroup determination

To determine the most appropriate outgroup available for our data, we reconstructed trees with the most likely outgroups based on Lockyer *et al.* (2003) and chose the sister group to the paramphistomoids (ingroup). Species from the following nine families were used from 12 digenean mitochondrial genomes for maximum-likelihood (ML) analysis: *Dicrocoelium dendriticum* (NC_025280), *Fasciola gigantica* (NC_024025), *P. cervi* (NC_023095), *Opisthorchis felineus* (NC_011127), *Clonorchis sinensis* (NC_012147), *Orthocoelium streptocoelium* (NC_028071), *Echinostoma hortense* (NC_028010), *Fischoederius elgonatus* (NC_028001), *P. westermani* (NC_027673), *Eurytrema pancreaticum* (NC_026916), *F. hepatica* (NC_002546) and *Ogmocotyle sikae* (NC_027112).

### Sequence alignment and phylogenetic analyses

Phylogenetic analyses were done with *cox*1 and ITS2 sequences using ML and Bayesian interference (BI). The analysis included four specimens from NCBI-GenBank for *cox*1 and 43 for ITS2 (Table 2). Non-identical haplotypes of *cox*1 and ITS2 sequences were aligned by eye and edited in MEGA6 (Tamura *et al.* 2013). A total of 690 bases were used for cox1 alignment and 385 bases for ITS2 alignments. Sequences generated in this study were submitted to GenBank (Table 2). ML analyses used PAUP* 4·0 b10 (Wilgenbusch and Swofford, 2003) and BI analyses were carried out using MrBayes (v 3.12) (Ronquist and Huelsenbeck, 2003). MrModeltest 2·0 (Nylander, 2004) was used to find the best fit model of substitution for BI and ML for both genes. Heuristic searchers were utilized for ML analyses (excluding the third codon for *cox*1) and 100 bootstrap replicates were run for each dataset. For BI analyses of the *cox*1 dataset (excluding the third codon for *cox*1), the parameters were: nst = 6, rates = invgamma and ngammacat = 4. Four heated chains were run simultaneously for 1 000 000 generations. For BI analyses of the ITS2 dataset, the parameters were: nst = 6, rates = gamma and ngammacat = 4. Four heated chains were run simultaneously for 1 400 000 generations. In both datasets, the trees were sampled every 100 cycles, and the first 25% of trees with pre-asymptotic likelihood scores were discarded as burn-in. A number of generations were determined sufficient because the s.d. dropped below 0·01 at the end of the runs.

Nucleotide substitution saturation at the third codon was tested in DAMBE5 (Xia, 2013) for *cox*1. Uncorrected pairwise distance values were calculated in MEGA6 (Tamura *et al.* 2013). Data were summarized within and between groups (Tables 3 and 4). We used similar criteria of other studies that used a *P*-distance value >5% difference with *cox*1 and *nd*1 mtDNA markers and >1·0% for ITS to indicate separate species (Vilas *et al.* 2005; Brant and Loker, 2009; Detwiler *et al.* 2010).

**Table 3.** Intra- and interclade *P-* distance values of *cox*1 amplified from paramphistomoids from Kenya

Clade	*N*	1	2	3	4	5	6	7	8	9	10	11	12	13	14	15	16
1. Clade 1	1	–															
2. Clade 2	17	0·185	**0·011**														
3. Clade 3	2	0·199	0·108	**0·003**													
4. Clade 4	5	0·175	0·127	0·132	**0·003**												
5. Clade 5	4	0·166	0·131	0·143	0·126	**0·010**											
6. Clade 6	1	0·157	0·155	0·162	0·133	0·120	–										
7. Clade 7	1	0·164	0·165	0·158	0·134	0·129	0·098	–									
8. Clade 8	1	0·167	0·146	0·163	0·132	0·128	0·062	0·105	**–**								
9. Clade 9	13	0·158	0·138	0·148	0·124	0·109	0·061	0·099	0·063	**0·010**							
10. Clade 10	2	0·167	0·155	0·160	0·138	0·133	0·126	0·140	0·126	0·128	**0·000**						
11. Clade 11	2	0·177	0·155	0·175	0·164	0·151	0·152	0·155	0·155	0·155	0·140	**0·001**					
12. Clade 12	30	0·171	0·156	0·157	0·138	0·122	0·135	0·144	0·145	0·136	0·130	0·160	**0·009**				
13. Clade 13	9	0·151	0·164	0·169	0·149	0·138	0·132	0·131	0·140	0·123	0·141	0·167	0·123	**0·012**			
14. Clade 14	2	0·158	0·162	0·176	0·144	0·131	0·130	0·126	0·132	0·121	0·138	0·145	0·129	0·088	**0·004**		
15. Clade 15	8	0·161	0·170	0·170	0·150	0·124	0·121	0·135	0·127	0·122	0·134	0·165	0·130	0·098	0·109	**0·010**	
16. Clade 16	22	0·165	0·151	0·179	0·145	0·130	0·142	0·132	0·130	0·140	0·142	0·157	0·131	0·100	0·119	0·111	**0·013**

Values in bold are intraclade divergences. Note that “–” indicates only a single specimen was collected and within distances could not be calculated.

**Table 4.** Intra- and interclade *p-*distance values of ITS2 amplified from paramphistomoids from Kenya

Clade	*N*	1	2	3	4	5	6	7	8	9	10	11	12	13	14	15	16
1. Clade 1	1	–															
2. Clade 2	17	0·063	**0·003**														
3. Clade 3	2	0·065	0·009	**0·000**													
4. Clade 4	5	0·064	0·009	0·016	**0·005**												
5. Clade 5	4	0·061	0·010	0·015	0·011	**0·002**											
6. Clade 6	1	0·077	0·042	0·045	0·043	0·038	**–**										
7. Clade 7	1	0·073	0·038	0·042	0·039	0·034	0·004	**–**									
8. Clade 8	1	0·073	0·038	0·042	0·039	0·034	0·004	0·001	**–**								
9. Clade 9	13	0·075	0·040	0·044	0·042	0·036	0·007	0·003	0·003	**0·003**							
10. Clade 10	2	0·068	0·017	0·023	0·018	0·014	0·040	0·036	0·036	0·039	**0·003**						
11. Clade 11	2	0·058	0·018	0·025	0·019	0·015	0·041	0·038	0·038	0·040	0·019	**0·006**					
12. Clade 12	30	0·067	0·036	0·042	0·038	0·034	0·050	0·046	0·046	0·048	0·038	0·021	**0·003**				
13. Clade 13	9	0·061	0·021	0·027	0·022	0·018	0·042	0·038	0·038	0·040	0·025	0·018	0·026	**0·003**			
14. Clade 14	2	0·057	0·022	0·029	0·023	0·019	0·035	0·031	0·031	0·034	0·026	0·017	0·025	0·006	**0·001**		
15. Clade 15	8	0·060	0·020	0·026	0·021	0·017	0·038	0·034	0·034	0·037	0·024	0·015	0·023	0·004	0·003	**0·001**	
16. Clade 16	22	0·061	0·027	0·033	0·028	0·023	0·040	0·035	0·035	0·038	0·030	0·021	0·029	0·011	0·004	0·007	**0·003**

Values in bold are intraclade divergences. Note that “–” indicates only a single specimen was collected and within distances could not be calculated.

## RESULTS

### Samples

Paramphistomoid adults were collected from three species of ruminants and cercariae and/or rediae were collected from four different genera of planorbid snails (*Biomphalaria, Bulinus, Ceratophallus, Segmentorbis*) from ten localities in central and west Kenya (Tables 1 and 2). Paramphistomoid cercariae were not found in other snail species examined (*Melanoides tuberculata, Radix natalensis, Physa acuta* and *Bellamya unicolor*). Ruminants were typically heavily infected, and often hundreds of adult worms could be quickly collected per host. From our samples collected, we examined and sequenced 79 adult and 41 cercariae specimens (120 total specimens) that represented obvious variants. To facilitate sampling if a large numbers of adult worms were acquired from a single host, we separated them by differences in adult host morphology (size and presence of a pouch or a genital sucker). To further assure collection of a diversity of specimens, we sampled both adult worms and rediae/cercariae from different localities

### Outgroup determination

With the diversity of sequence data available in GenBank, our analysis revealed that *O. sikae* (Notocotylidae) is more closely related to paramphistomoids than members of Echinostomatidae or Fasciolidae used as outgroups for other paramphistomoid molecular phylogenies (Lotfy *et al.* 2010; Shylla *et al.* 2011; Ghatani *et al.* 2012). For phylogenetic analyses of both genes, we used three species of notocotylids as outgroup taxa.

### Cox1 phylogenetic analyses and pairwise distance divergences

**Fig. 1.**
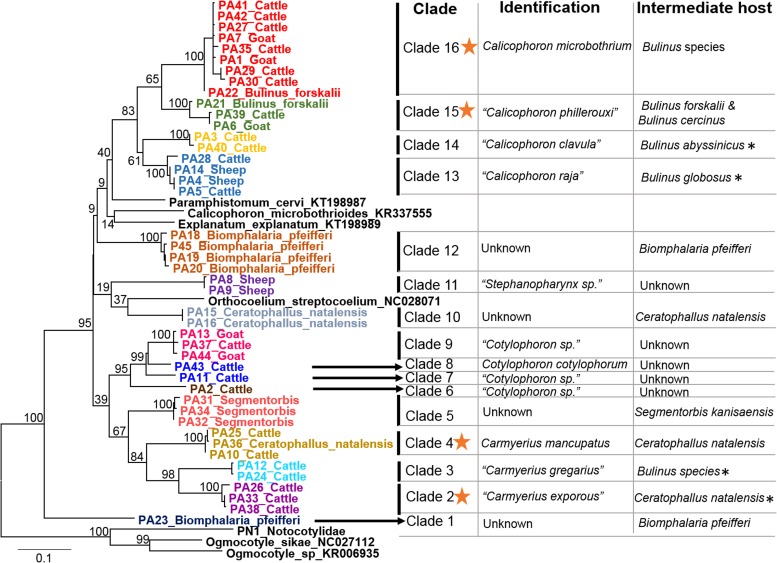
Phylogenetic relationships of 44 samples of paramphistomoids from this study and from GenBank based on *cox*1 (690 bp) sequences inferred from ML (bootstrap values) analysis. Specimens are named based on sample name, the host it was collected from and are colour coded based on intraclade *P-*distance values <1·3% and interclade values >6·5%. An orange star represents clades where we matched cercariae and adult sequences. Identifications were made based on GenBank sequences and on the species descriptions in the literature (parentheses). An (*) denotes intermediate host use from studies in the literature that have not been sequenced confirmed.

**Fig. 2.**
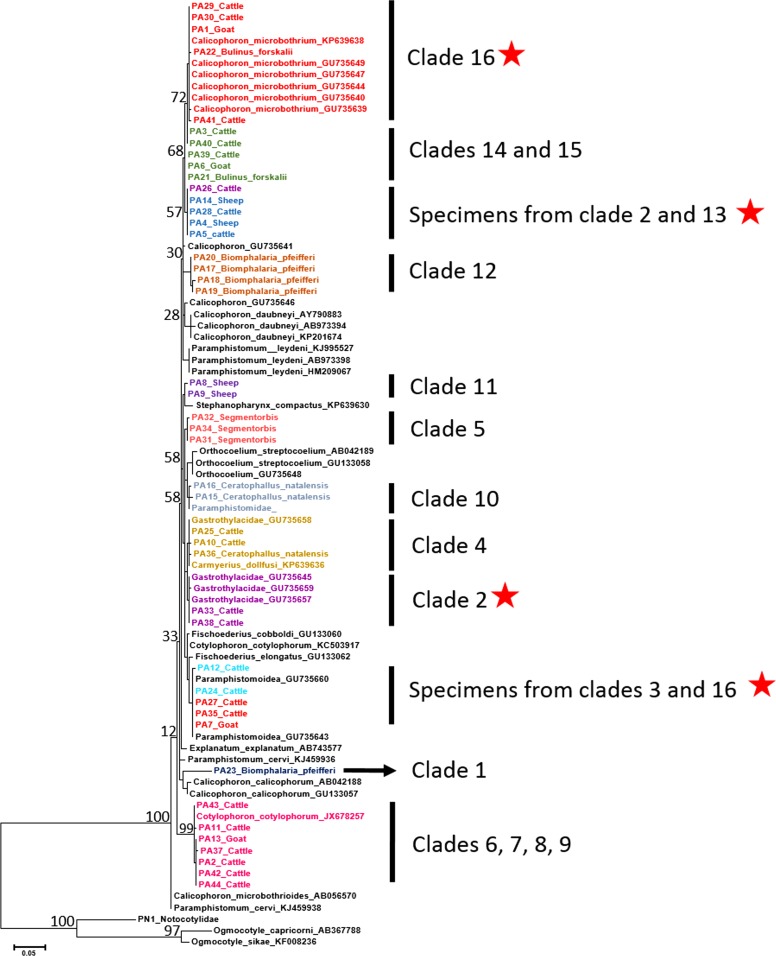
Phylogenetic relationships of 44 samples of paramphistomoids from this study and from GenBank based on ITS2 (385 bp) sequences inferred from ML (bootstrap values) analysis. Specimens are named based on sample name, the host it was collected from, and colour coded based on clade designation from *cox*1 distance values. A red star represents clades where we have found evidence of putative hybrids. Adjacent to these indicated clades, are clade numbers that correspond to the same specimens and clade numbers as appearing on the *cox*1 tree (Fig. 1).

In general, trees were first constructed incorporating all 120 specimens (Supplementary Figs. S1 and S2). Because some clades were represented by multiple specimens (haplotypes with a 1–4 bp difference for *cox*1) we reduced the number of specimens per clade to simplify the trees for display purposes (Figs. 1 and 2). Many of the deeper nodes were not supported; however, the trees nonetheless provided a useful way to visualize the overall diversity of specimens found, and to provide comparisons with available systematic treatments. The specific clades identified (names next to the bolded black vertical lines) on the *cox*1 tree represent conspecifics (Fig. 1).

Partial sequences of *cox*1 (690 bp) were obtained for all 120 samples (Supplementary Fig. S1). ML and BI (Supplementary Fig. S3) trees were created for the *cox*1 alignment, and the ML tree is shown (Fig. 1). MrModeltest 2·3 selected the GTR + I + G model of nucleotide substitution. Based on bootstrap and posterior probabilities in Table 3, 16 distinct *cox*1 clades were identified among Kenyan specimens and are portrayed alongside the tree in Fig. 1 (vertical black lines or arrows). We used genetic distance data to determine if a clade was comprised more than one species. A single species was of determined for specimens with genetic distance values <1·3%, and species were designated as distinct when genetic distance values were >6·2% (Table 3). Most interclade pairwise distance values were >10·0% and they ranged up to 19·9%. These same clade numbers or scientific names were also used adjacent to the ITS2 tree in Fig. 2.

### ITS2 phylogenetic analyses and pairwise distance divergences

For ITS2, sequences were obtained from all 120 samples and our phylogenetic analyses also included 46 samples from GenBank (Supplementary Fig. S2). The ITS2 alignment included 61 bp of 5·8S, 283 bp of ITS2 and 46 bp of 28S. The average intraclade pairwise distance was 0·30% and the average interclade pairwise distance was 3·9% (Table 4). MrModeltest 2·3 selected the GTR + G model of nucleotide substitution for ITS2. Both BI and ML analyses were run using 33 or 46, respectively, additional relevant species sequences from GenBank, with the ML tree shown (Figs 2 and Fig S4). Not surprisingly, the degree of resolution provided by phylogenetic analysis of ITS2 sequences was not high given the more conservative rate of change of this widely used nuclear gene marker (Locke *et al.* 2010). Based on ML and BI analyses, 12 ITS2 clades were identified among our Kenyan specimens (Fig. 2 and Supplementary Fig. S4). Intraclade genetic distance values were <0·6%, and interclade genetic distance values were >1·0%.

### Further comparisons of the cox1 and ITS2 datasets

*Cox*1 and ITS2 trees did not conflict, but the ITS2 trees did not have as much support for the deeper nodes as *cox*1 (Figs 1 and 2). All 12 clades from ITS2 were represented in the *cox*1 dataset. The *cox*1 genetic distance data enabled differentiation among some of the worms clustered with *Cotylophoron cotylophorum* in the ITS2 dataset, and also clearly differentiated clades 14 and 15 (Fig. 2).

In three cases (clades 4, 10 and 16), *cox*1 sequence matches (<1·3%) were obtained between worms from ruminants and cercariae from snails (Fig. 1, orange stars). Clade 2 matched an ITS2 sequence from GenBank of cercariae from *Ceratophallus natalensis*, thus also confirming the intermediate host for this clade (Fig. 1). In four cases (clades 1, 5, 10 and 12), sequences were found from cercariae with no matches from adult worms for either sequence (Fig. 1). In at least five cases (PA7, PA26, PA27, PA35 and PA42), the ITS2 nuclear sequences obtained clustered in different clades than what is seen in the *cox*1 trees (clades highlighted with red star in Fig. 2). These samples appear to have nuclear mitochondrial discordance (NMD) and are identified as worms with likely hybrid ancestry (see discussion).

### Provisional identification of the paramphistomoids

**Fig. 3.**
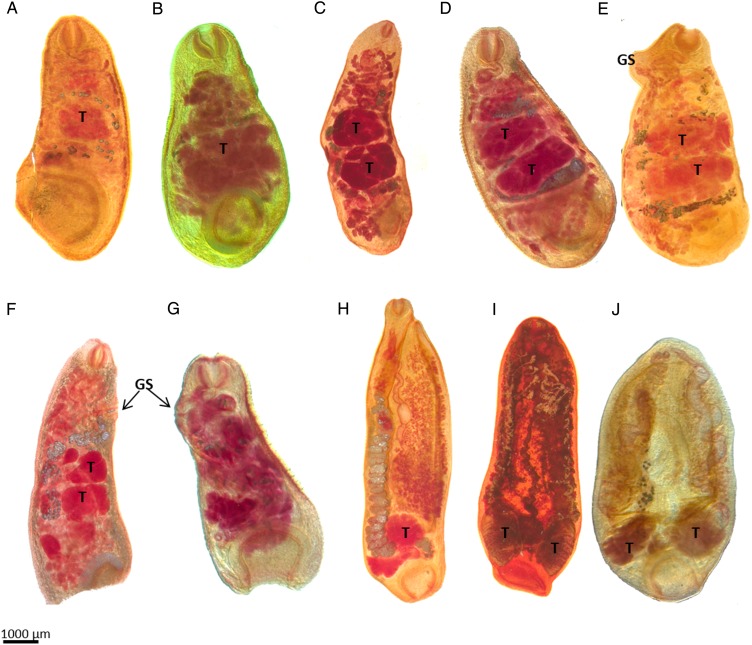
Sections of adult paramphistomoids collected from domestic ruminants in Kenya and their provisional identifications. (A) *Calicophoron phillerouxi*, (B) *Calicophoron raja*, (C) *Calicophoron clavula*, (D) *Calicophoron microbothrium*, (E) *Cotylophoron* sp., (F) *Cotylophoron cotylophorum*, (G) *Cotylophoron* sp., (H) *Carmyerius exporous*, (I) *Carmyerius gregarius*, (J) *Carmyerius mancupatus*. Note that the photographed specimens represent sections of adults, and presence of some organs like the testes (T) or genital sucker (GS) are indicated. For the genus *Carmyerius*, a ventral pouch was present, but is not visible in the sections chosen for presentation.

Provisional identifications were based on the paramphistomoid systematics literature (Eduardo, 1983; Sey, 1991; Jones, 2005*b*, *c*, *d*) pertaining to intermediate or definitive host use, and descriptions of adult worms in comparison to our mounted adult specimens (Table 5, Fig. 3). Some of the sequences we obtained matched sequences from named species in GenBank, and in those cases the names we provide here are the ones from GenBank (clades 4, 8 and 16). Four clades were represented only by cercariae and did not match any sequences derived from adult worms in this study or from GenBank. These included two clades from *B. pfeifferi* (clades 1 and 12), one from *Segmentorbis kanisaensis* (clade 5) and one from *C. natalensis* (clade 10). Our 16 clades represented three different families of Paramphistomoidea: Gastrothylacidae, Paramphistomidae and Stephanopharyngidae. Species names in quotation marks in Fig. 1 were assigned based on our morphological identification from species descriptions.

**Table 5.** Provisional identification of the paramphistomoids was based on species descriptions and intermediate host use from the literature and on position in phylogenetic trees

Clade	Provisional identification	Stage	Ventral pouch	Acetabulum type	Genital sucker	Known intermediate hosts	Hosts from this study	References
1	Unknown	C	n/a	n/a	n/a	n/a	*B. pfeifferi*	(Sey, 1991; Jones, 2005*a*)
2	*Carmyerius exporus*	C, A	Yes	*Carmyerius*	No	*Ceratophallus natalensis*	*C. natalensis* and cattle	(Dinnik, 1965; Sey, 1991; Jones, 2005*c*)
3	*Carmyerius gregarius*	A	Yes	*Carmyerius*	No	*Bulinus* species	Cattle	(Looss, 1896; Sey, 1991)
4	*Carmyerius mancupatus*	C, A	Yes	*Gastrothylax*	No	*Ceratophallus natalensis*	*C. natalensis,* cattle, sheep and goats	(Gretillat, 1964; Dinnik, 1965; Sey, 1991; Jones, 2005*c*)
5	Unknown	C	n/a	n/a	n/a	n/a	*S. kanisaensis*	(Sey, 1991; Jones, 2005*c*)
6	*Cotylophoron* sp.	A	No	*Cotylophoron*	Yes	Unknown	Cattle	(Sey, 1991; Jones, 2005*b*)
7	*Cotylophoron* sp.	A	No	*Cotylophoron*	Yes	Unknown	Cattle	(Sey, 1991; Jones, 2005*b*)
8	*Cotylophoron cotylophorum*	A	No	*Cotylophoron*	Yes	Unknown	Cattle	(Sey, 1991; Eduardo, 1983; Jones, 2005*b*)
9	*Cotylophoron* sp.	A	No	*Cotylophoron*	Yes	Unknown	Cattle, sheep and goats	(Sey, 1991; Jones, 2005*b*)
10	Unknown	C	n/a	n/a	n/a	*Ceratophallus natalensis*	*C. natalensis*	(Sey, 1991; Jones, 2005*a*)
11	*Stephanopharynx* sp.	A	No	*Stephanopharynx*	No	Unknown	Sheep	(Sey 1991; Jones, 2005*d*)
12	Unknown	C	n/a	n/a	n/a	n/a	*B. pfeifferi*	(Sey, 1991; Jones, 2005*a*)
13	*Calicophoron raja*	A	No	*Calicophoron*	No	*Bulinus globosus*	Cattle, sheep and goats	(Dinnik and Dinnik, 1954; Eduardo, 1983; Sey, 1991)
14	*Calicophoron clavula*	A	No	*Calicophoron*	No	*Bulinus abyssinicus*	Cattle	(Sobrero, 1962; Eduardo, 1983; Sey, 1991)
15	*Calicophoron phillerouxi*	C, A	No	*Calicophoron*	No	*Bulinus forskalii*	*B. forskalii,* cattle, sheep and goats	(Dinnik, 1961; Eduardo, 1983; Sey, 1991)
16	*Calicophoron microbothrium*	C, A	No	*Calicophoron*	No	*Bulinus* species	*B. forskali,*cattle, sheep and goats	(Dinnik and Dinnik, 1954; Eduardo, 1983; Sey, 1991)

Cercariae (C), adults (A) and their associated hosts are listed. Ventral pouch, acetabulum type and genital sucker were useful morphological features for genus and species placement.

## DISCUSSION

Paramphistomoid flukes are speciose in sub-Saharan Africa, reflective of the presence there of many mammal species, particularly wild and domestic ruminants. These flukes are also ubiquitous and can have a high prevalence among domestic ruminants reaching 100% in some villages (Chingwena *et al.* 2002*a*; Nzalawahe *et al.* 2015). During our sampling of Kenyan slaughterhouses we found up to 90% of the domestic ruminants infected, and many individual animals harboured hundreds of adult worms. Of the many adult worm and cercariae samples collected, we further investigated 120 samples (79 adult worms and 41 cercariae) determined most likely to be genetically distinctive. We found 16 distinct clades in three families of the Paramphistomoidea. For future comparisons, all of our specimens are available as vouchers at the Parasite Division, Museum of Southwestern Biology (MSB) or at the Kenyan Medical Research Institute (KEMRI).

Previous studies have used the easily obtained ITS2 sequence as a molecular marker to distinguish among paramphistomoid species (Itagaki *et al.* 2003; Rinaldi *et al.* 2005; Goswami *et al.* 2009; Lotfy *et al.* 2010; Sanabria *et al.* 2011; Ichikawa *et al.* 2013; Shylla *et al.* 2013; Ghatani *et al.* 2014; Dube *et al.* 2015). ITS2 is helpful for distinguishing paramphistomoid genera and differentiating more divergent species within a genus (Rinaldi *et al.* 2005; Ghatani *et al.* 2012). Because mitochondrial DNA accumulates substitutions more frequently than the internal transcribed spacers, it is more useful to differentiate among closely related species, particularly cryptic species (Blouin, 2002; Vilas *et al.* 2005; Locke *et al.* 2015), or to reveal intraspecific variation (Ghatani *et al.* 2014). Consequently, we used genetic distance values for *cox*1 sequence data as the primary means to delineate species. For *cox*1, interclade *P*-distance values were >6·2%, although the majority of pairwise comparisons were >10·0%. In contrast, intraclade pairwise divergence values were <1·3%. Other studies have used a *P*-distance value >5% difference with *cox*1 and *nd*1 mtDNA markers to indicate separate species (Vilas *et al.* 2005; Brant and Loker, 2009; Detwiler *et al.* 2010). Our data suggests that ITS2 should not be used alone to differentiate species for paramphistomoids.

We also examined the delineated clades with respect to where they grouped in either ML or BI phylogenetic analyses based on either *cox*1 or ITS2 sequences. In general, there was low bootstrap/posterior probability support for many of the deeper nodes in either ML or BI trees, suggesting that broader taxon sampling, along with sequencing of additional markers, is needed to more definitively support or refute the morphologically based systematic framework developed for paramphistomoids (Sey, 1991; Jones, 2005*a*). The phylogenetic trees were useful, however, in providing preliminary hypotheses for how the various clades were related to one another (see the paragraph below). Relative to other paramphistomoid molecular phylogenetic studies involving specimens from African ruminants and snails, we recovered five out of the six previously reported taxa from Kenya, Egypt and Tanzania noted by Lotfy *et al.* (2010), three of the three identified taxa from Zimbabwe, Zambia and Botswana (Dube *et al.* 2015) and one of the two identified taxa from Algeria (Titi *et al.* 2014). The extent of overlap among specimens recovered from all four studies suggests that at least some of the species have broad distributions in Africa. Additional sampling is needed to provide a more comprehensive picture of African paramphistomoid diversity, particularly from Central and West Africa.

The phylogenetic trees provided support for anatomically based taxon delineations as four clades identified as *Calicophoron* grouped together, as did three clades of *Carmyerius* and four clades of *Cotylophoron*. Furthermore, worms in the Stephanopharyngidae (*Stephanopharynx*) formed a clade, as did presumptive members of the Gastrothylacidae. However, all presumptive members of the Paramphistomidae did not group together. It is possible that this is a paraphyletic group or certain genera, such as *Cotylophoron* belong in a different family. Clade 1 is quite divergent from the other specimens discussed and it is possible it represents a different family or superfamily. The trees also show some incongruences between nuclear and mitochondrial sequences (discussed further below).

With respect to host use, specimens from a particular clade were reported from the same snail host species or genus. Also, different clades that group together tend to share the same genus of snail host (*Calicophoron*, in clades 13–16, in *Bulinus*) or snail genera in related tribes (*Carymerius* in clades 2, 3 and 5 in *Segmentorbis* and *Ceratophallus*). For 10 of 11 clades for which snail host usage could be identified, those snails belong in the family Planorbidae. Snail host use may thus have had an important impact on paramphistomoid diversification, which has also been suggested for other digenean groups (Brant and Loker, 2013). In only one instance have we found cercariae that we have assigned to the same clade (clade 10) that derive from two different snail genera: cercariae from *C. natalensis* collected from this study and cercariae from *Biomphalaria sudanica* collected by Lotfy *et al*. (2010). Many other digenean groups also indicate high first intermediate host specificity (Shoop, 1988; Donald *et al.* 2004; Detwiler *et al.* 2010; Brant and Loker, 2013). By contrast, adult worms of a particular clade were often recovered from more than one definitive host species, and we recovered up to three different taxa of paramphistomoids from an individual bovine.

Sequence data derived from life cycle stages from different hosts provide an important alternative way to piece together the complex life cycles of digeneans, especially when experimental exposures are not possible (Chibwana *et al.* 2015). We provide supportive evidence for the life cycles of four of our identified clades (Fig. 1) by matching genetic sequences (<0·6% for ITS2 and <1·3% *cox*1) collected from cercariae and adults: (1) ITS2 sequences from cercariae from *C. natalensis* (GU735645) collected in Kenya grouped with sequences from adult worms we recovered from cattle (clade 2), provisionally identified as *Carmyerius exporous* (Dinnik and Dinnik, 1960). (2) Cercariae (clade 4) we collected from *C. natalensis* matched adults collected in this study as well as two adults from Botswana (KP639636) and Kenya (GU735658) identified as *Carmyerius dollfusi* by Dube *et al.* (2015). The latter species was synonymized with *C. mancupatus* (Sey, 1991), a species known to be transmitted by *C. natalensis* (Dinnik, 1965). (3) Sequences from seven adults we obtained (clade 15) matched sequences collected from a cercariae sample from *B. forskalii*. We provisionally identified the adults as *C. phillerouxi*, which is known to be transmitted by *B. forskalii* (Dinnik, 1961). (4) Lastly, two cercariae samples we collected from *B. forskalii* matched with 23 adults collected in this study, and with one cercariae sample from *B. forskalii* and 18 adults in GenBank, all of which were identified as *C. microbothrium* (clade 16). As the host record and sequence databases grow, the probabilities that more matches will be found also increases, providing a way forward in working out life cycles that will help offset increasing difficulties in doing so with more conventional experimental infections.

The most common paramphistomoid genus we collected was *Calicophoron* (40 out of the 120 specimens examined), and the most abundant species was *Calicophoron microbothrium* which is transmitted by bulinid snails. This species is the most geographically widespread paramphistome in Africa, its presence confirmed with molecular markers from Egypt, Kenya, Tanzania, Zambia, Zimbabwe, South Africa, Algeria and Botswana (Lotfy *et al.* 2010; Titi *et al.* 2014; Dube *et al.* 2015). Given the difficulties in discriminating this species from others based on morphology alone, the broad geographic distribution, and the diversity of different bulinid snails reported as hosts, this species is a good candidate for further inspection as a possible complex of cryptic species. Presently the best sequence available to evaluate this possibility is *cox*1, but most of the data in the literature thus far for this species are for ITS2. Our ML analysis based on 354 bp of ITS2 (figure not shown) suggests there are distinct clades among the samples identified as *C. microbothrium* in GenBank, with an average distance among them of 0·75%. Other sequence markers are needed to determine if *C. microbothrium* is a complex of cryptic species, and how well differentiated they prove to be from the other *Calicophoron* clades (13–15) identified in this study.

We found some specimens with discordant nuclear and mitochondrial sequences, consistent with the possibility of hybrid origins (red stars, Fig. 2). For example, two samples (PA12 and PA24) grouped with *C. microbothrium* in the ITS2 trees, but fell in their own clade (3) in the *cox*1 trees. PA12 and PA24 were also morphologically distinct from *C. microbothrium*, being provisionally identified as members of the gastrothylacid genus *Carmyerius*. As we have noted, multiple species of paramphistomoids are frequently recovered from a single ruminant host, creating circumstances conducive for potential hybridization. The putative parental species and hybrids (PA7, PA12, PA24 PA27, PA35) all use *Bulinus* as intermediate hosts. It seems possible that the likelihood of successful hybridization would be increased if both parental species use the same genus or species of intermediate host, if as appears intermediate host use is more specific than definitive host use among the paramphistomoids. Other examples of sequence discordance in digeneans also involve groups with closely related species that can hybridize, and that share snail hosts, such as with some species of fasciolids and schistosomes (Steinauer *et al.* 2008; Peng *et al*. 2009). Further studies using microsatellite markers or RADSeq technology will be needed to verify a hybrid origin for paramphistomoids with discordant sequences.

Members of the basommatophoran family Planorbidae are the most common intermediate hosts transmitting paramphistomoids in Kenya, although snails of the Family Lymnaeidae have also been identified as hosts for paramphistomoids in East Africa (Sey, 1991). The snail hosts for some of the clades we have identified such as clades 3, 6, 7, 8 (*C. cotylophorum*), 9 and 11 (*Stephanopharynx* sp.) are unknown or require additional sequence-based verification. *Bulinus* snails, with an ancient history and diversification in Africa (Van Damme 1984; Brown, 1994; De Groeve, 2005), are particularly prominent as African paramphistomoid hosts (Sey, 1991). By contrast, *Biomphalaria* supports fewer paramphistomoid species and has a much shorter evolutionary history in Africa, with estimates ranging from <1–5 mya (million years ago) (Woodruff and Mulvey, 1997; Campbell *et al.* 2000; DeJong *et al.* 2001). It is noteworthy that clade 1, which is known only from cercariae from *B. pfeifferi*, is one of the most divergent clades we recovered. Clade 1 cercariae are also much larger than the other paramphistomoid cercariae we recovered (about 2·0× longer in combined body and tail length). This raises a possibility that the diversification of paramphistomoids is more recent than the longer evolutionary history of *Bulinus* in Africa might suggest. More data are needed to resolve the phylogenetic position of this and other paramphistomoid clades, including those found in non-ruminant species.

In Kenya, *Bulinus globosus, B. nasutus, B. africanus, B. tropicus, B. forskalii* and *Biomphalaria pfeifferi*, are known to transmit paramphistomoids as well as ruminant and/or human schistosomes (Southgate *et al.* 1989; Brown, 1994). The overlap in use of snail hosts creates opportunities for distinctive interactions between the two common digenean groups. For example, in Kenya, Southgate *et al.* (1989) found that *Bulinus tropicus* was capable only of supporting the development of *Schistosoma bovis* to production of cercariae if it was first exposed to *C. microbothrium.* Similarly, in South America, *Biomphalaria oligoza* and *Biomphalaria orbignyi* are naturally resistant to *S. mansoni*, but become susceptible to *S. mansoni* if first exposed to *Zygocotyle lunata* (Spatz *et al.* 2012). Paramphistomoids can also have the opposite influence on the success of other digeneans during co-infections. For example, as compared to snails exposed only to *F. hepatica*, significantly fewer *Pseudosuccinea columella* produced *F. hepatica* cercariae if first exposed to *Calicophoron daubneyi* and then later exposed to *F. hepatica* (Dreyfuss *et al.* 2016).

This study has shown that even in a fairly circumscribed area within one East African country that a considerable diversity of paramphistomoid flukes is present and that several of these fluke species are abundantly represented. Paramphistomoids are of veterinary interest because of their ubiquitous presence in herds of cattle, sheep and goats that are routinely watered in natural habitats where the presence of susceptible species of snails ensures their transmission. Whether the species we have encountered have long parasitized domestic livestock or represent recent acquisitions from the region's many wild ruminants is an interesting question for future study. Studies currently underway in Kenya indicate that paramphistomoid infections are very common in some snail populations, so much so that they may represent significant impediments to the ongoing transmission of schistosomes using the very same snail hosts in the same aquatic habitats (Laidemitt M.R., personal communication, 2016). Furthermore, the spectra of freshwater snails used by these two common digenean groups are broadly overlapping, further increasing the likelihood that interesting interactions and accommodations have been made over evolutionary time. It will be interesting to more fully ascertain how these two major groups of digeneans influence one another's abundance. It is clear though that the domestication of livestock ensures that both paramphistomoid and schistosome (both human and ruminant schistosome species) life cycles are perpetuated side-by-side in the same habitats year after year. Livestock domestication may well prove to have had multiple downstream effects – mediated by the digeneans of livestock – on the present-day transmission of the all-too-common human blood flukes of sub-Saharan Africa.

## Supplementary material

For supplementary material accompanying this paper visit http://dx.doi.org/10.1017/S0031182016001827. click here to view supplementary material
