# Supplementary figures and images for: Loads of trematodes: discovering hidden diversity of paramphistomoids in Kenyan ruminants
Source: Parasitology. 2016 Oct 20;144(2):131–47. doi: 10.1017/S0031182016001827 (PMC5300004; doi:10.1017/S0031182016001827)

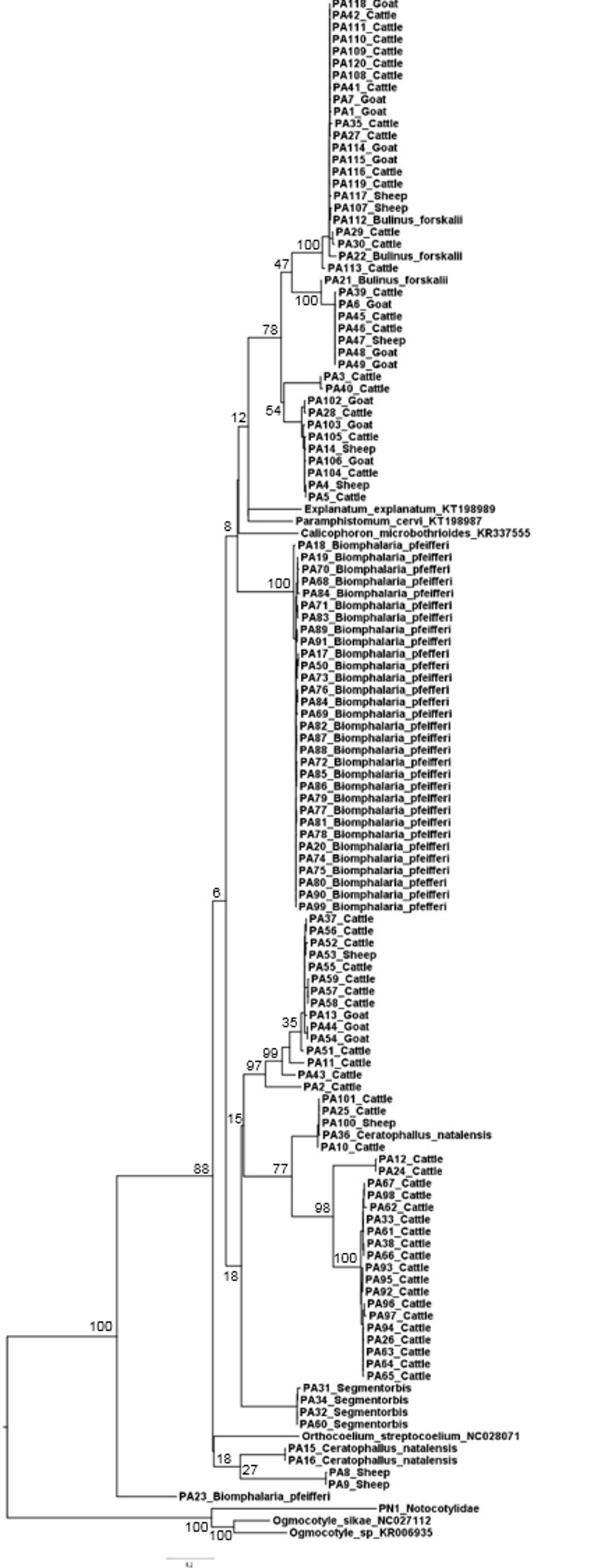

Supplement: Supplementary file 1 [file S0031182016001827sup001.zip › S0031182016001827sup001/S0031182016001827sup001.tif]

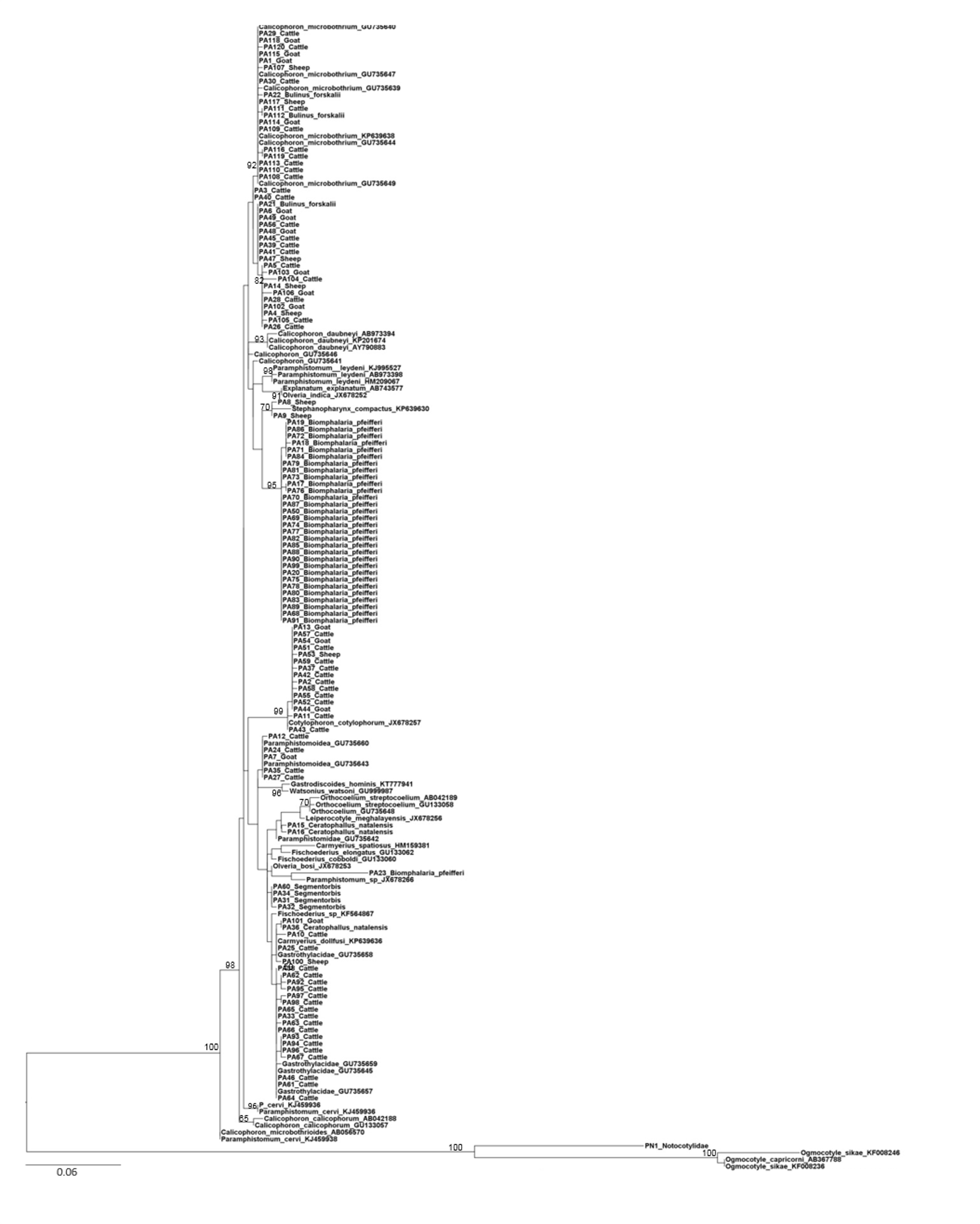

Supplement: Supplementary file 1 [file S0031182016001827sup001.zip › S0031182016001827sup001/S0031182016001827sup002.tif]

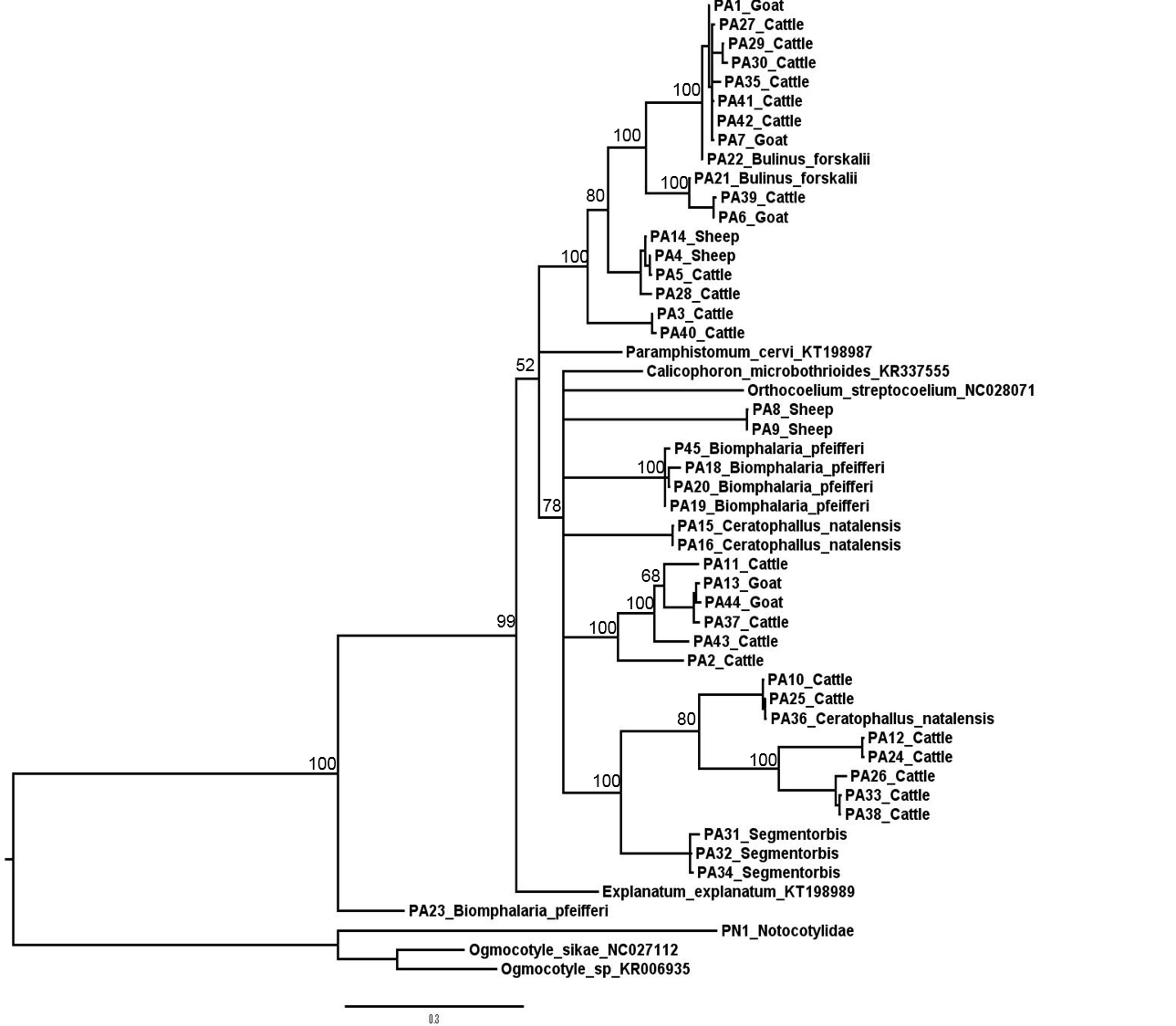

Supplement: Supplementary file 1 [file S0031182016001827sup001.zip › S0031182016001827sup001/S0031182016001827sup003.tif]

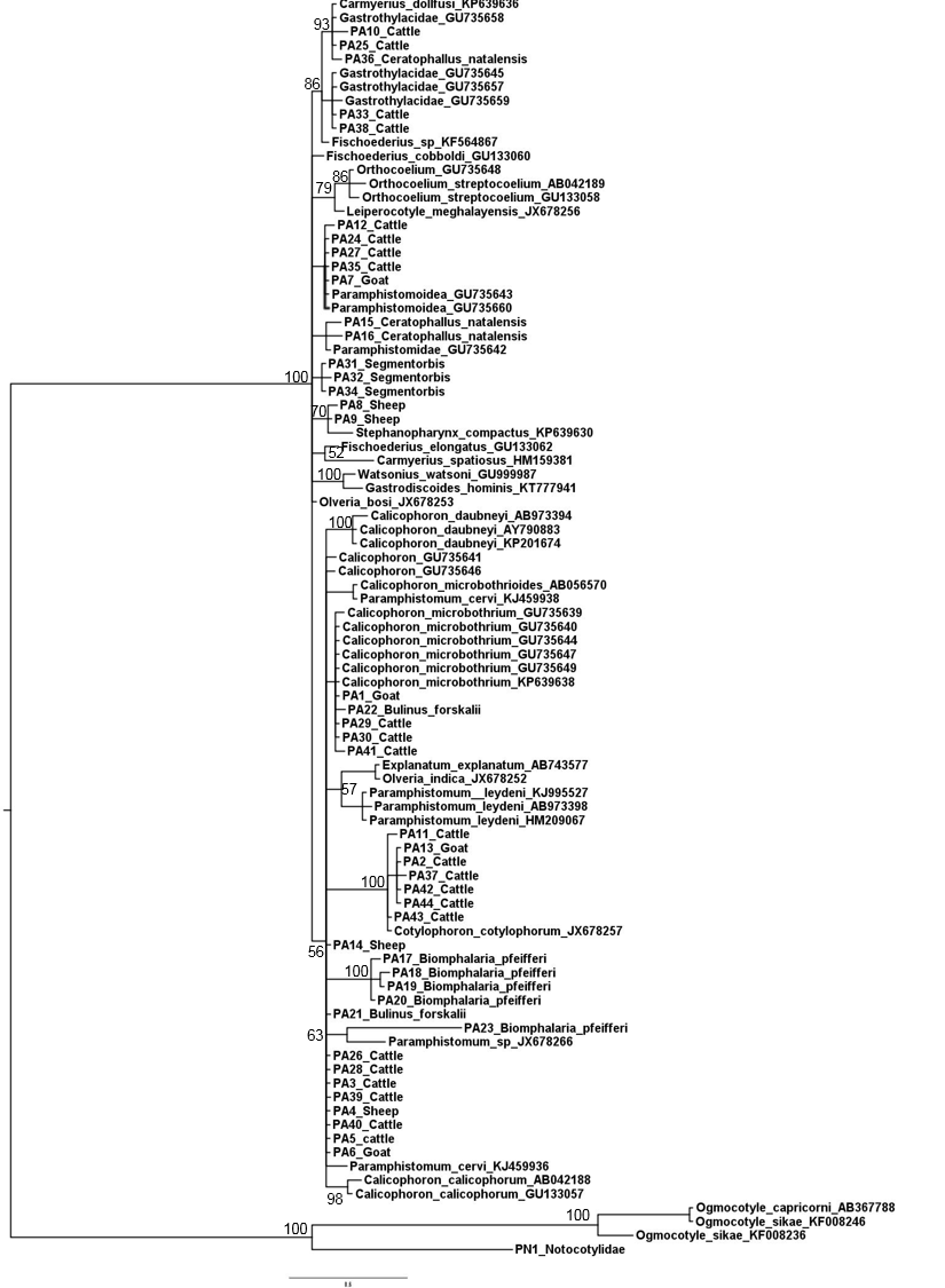

Supplement: Supplementary file 1 [file S0031182016001827sup001.zip › S0031182016001827sup001/S0031182016001827sup004.tif]
